# Transgene Expression and Bt Protein Content in Transgenic Bt Maize (MON810) under Optimal and Stressful Environmental Conditions

**DOI:** 10.1371/journal.pone.0123011

**Published:** 2015-04-08

**Authors:** Miluse Trtikova, Odd Gunnar Wikmark, Niklaus Zemp, Alex Widmer, Angelika Hilbeck

**Affiliations:** 1 ETH Zurich, IBZ, Plant Ecological Genetics, Universitaetstrasse 16, 8092 Zurich, Switzerland; 2 Genok—Centre for Biosafety, 9294 Tromso, Norway; Mayo Clinic Arizona, UNITED STATES

## Abstract

Bt protein content in transgenic insect resistant (Bt) maize may vary between tissues within plants and between plants growing under different environmental conditions. However, it is unknown whether and how Bt protein content correlates with transgene expression, and whether this relationship is influenced by stressful environmental conditions. Two Bt maize varieties containing the same transgene cassette (MON 810) were grown under optimal and stressful conditions. Before and during stress exposure, the upper leaves were analysed for transgene expression using quantitative RT-PCR and for Bt content using ELISA. Under optimal conditions there was no significant difference in the transgene expression between the two investigated Bt maize varieties whereas Bt protein content differed significantly. Transgene expression was correlated with Bt protein content in only one of the varieties. Under stressful environmental conditions we found similar transgene expressions as under optimal conditions but Bt content responded differently. These results suggest that Bt content is not only controlled by the transgene expression but is also dependent on the genetic background of the maize variety. Under stressful conditions the concentration of Bt protein is even more difficult to predict.

## Introduction

Genetic modification of crop plants often has the goal to engineer lines that express novel traits that cannot be introduced into the crop by conventional breeding. Such bioengineering efforts build on the expectation that target gene(s) conferring the desired trait, in association with suitable regulatory elements that are also part of the transgene construct, express the desired trait in a stable and reliable manner. This expectation remains to be evaluated, for example when the transgene is introduced into different genetic background (i.e. varieties) or when genetically modified (GM) plants are exposed to diverse environmental conditions.

One of the two most widely marketed GM traits worldwide is insect resistance, which is conferred by insecticidal toxins from *Bacillus thuringiensis* (Bt). This trait has been engineered into a number of crop plants, including maize and cotton. Maize containing insect resistance trait is currently grown on more than 47 million hectares worldwide [1], which represents about 27% of the global area planted with maize [2]. Bt maize cultivars derived from the MON810 event specifically contain a transgene cassette consisting of the cauliflower mosaic virus 35S promoter, thet e hsp70 intron and the *cry1Ab* gene endowing the resulting MON810 Bt maize plants with a resistance to lepidopteran pest species, particularly, the European corn borer, *Ostrinia nubilalis*.

It is generally expected that in commercial GM plants, transgenes are constitutively expressed at high levels and in all plant tissues and phenological phases [3]. Tight control over transgene expression and Bt protein content is important in light of concerns over the evolution of Bt toxin resistance in target insects [4]. In recent years, several studies have reported that the Bt protein concentration may vary within Bt plants (i.e. across tissues) and over growing seasons [5–7], while other authors reported that abiotic factors, such as nitrogen fertilization [8], soil quality and pesticide use [9] can affect Bt protein content. However, to the best of our knowledge, no study has been published to date that jointly investigated the relationship between Bt transgene expression and Bt protein content in transgenic Bt maize. In Bt cotton, this relationship has been investigated in a limited number of studies [10–12]. Olsen et al. [12] and Adamczyk et al. [10] found correlations between mRNA transcript levels of insect resistance transgene *cry1Ac* and Bt protein content, whereas Li et al. [11] observed no such relationship under salt stress. It therefore currently remains open what the relationship between Bt transgene expression and Bt protein content in GM crops is.

Establishing whether such a relationship exists in Bt maize and how it is affected by environmental conditions is an important question. In most countries where cultivation of Bt maize has been approved, this was done on the condition of installing an insect resistance management (IRM) program. One of the pillars of IRM is that plants contain high and stable levels of the Bt protein that are lethal not only to susceptible target insects but also to heterozygotes that carry one resistance allele (RS genotypes) [13]. The aim of IRM is to delay the evolution of resistance to Bt toxins in target pests which has been identified as a prime threat to the sustainability of Bt crops [4].

The aims of this study were to explore the relationship between Bt transgene expression and Bt protein content in two Bt maize varieties, and to experimentally test whether abiotic environmental stress conditions influence the relationship between transgene expression and protein content.

## Materials and Methods

### Plant material and treatments

Seeds of two Bt maize (MON 810) varieties (white Bt—PAN 6Q-321B and yellow Bt—PAN 6Q-308B) were sown into two litre plastic pots filled with the potting soil (Oekohum Topferde mit Kokos) and covered with a layer of gravel. Fifteen plants of each variety were first grown in the climate chambers under optimal conditions (16/8 L/D, 25/20°C, 50/65% rh, watered regularly). After six weeks, the plants were either kept further under optimal conditions or exposed to stressful environmental conditions for one week. The stressful conditions included a hot/dry treatment in a greenhouse: 16/8 L/D, temperature varied in a shade between 21–30°C and in a full sun reached up to 45°C, relative humidity varied between 39–67%, the plants were watered sparsely, 100 ml per pot on a daily basis. Or a cold/wet treatment was applied: 16/8 L/D, 16/13°C, 65/80% rh, waterlogged for 24 h, afterwards soil kept saturated with water. Before application of the stress treatment, the plants had on average 11 leaves and 7 collars. After one week of cold/wet and hot/dry stress, the plants had 11 leaves and 8 collars, whereas under optimal conditions they had on average 12 leaves and 8 collars.

### Plant sampling

The second upper fully developed leaves were sampled before the stressful conditions were applied. The tips of the leaves were cut off and seven about 4 cm^2^ pieces were cut next to each other avoiding the main leaf vein. Following this approach we could reduce the variation in Bt content within the single leaf [7]. The five leaf pieces assigned to be analysed for transgene expression were immediately frozen in liquid nitrogen and later stored at -80°C. The two leaf pieces collected to measure Bt content were kept on ice and later stored at -20°C. After one week of stressful conditions, the same plants were resampled. When possible, the samples were not collected from the same previously cut leaves. Instead, seven leaf pieces were cut from the leaves above or from newly formed leaves.

### qRT-PCR

RNA was extracted from 60 leaf samples (15 white Bt and 15 yellow Bt maize plants sampled before and during stress) using RNeasy Plant Mini Kit (Qiagen). RNA quality was checked on Bioanalyzer Agilent 2100 using Plant RNA Nano chips. Only samples with RIN higher than 5 were used for further analysis. RNA was treated with RDD Buffer (Qiagen) and DNAse (Qiagen) and inactivated with EDTA (Invitrogen). QuantiTect Reverse Transcription Kit (Qiagen), including the Wipeout Buffer, was used to produce cDNA. The samples were run in triplicates in a 11 μl reaction volume using TaqMan Gene Expression Master Mix (Applied Biosystems). The primers and probe sequences for *cry1Ab* transgene were kindly provided by A. Coll (Institut de Tecnologia ària (INTEA), Universitat de Girona). Additionally, four reference genes (*mep*, *ubcp*, *cul*, *lug*) as recommended by Manoli et al. 2012 [14] were chosen to normalize the qRT-PCR data. TaqMan primers and probes for reference genes were designed based on the sequences obtained from Maize Genetics and Genomics Database (http://www.maizegdb.org/) using Primer Express 3.0 software (Applied Biosystems). Amplification efficiencies were estimated using LinRegPCR software version 2012.3 [15] (S1 Table). The stability of the reference genes was assessed using geNorm and *cul* was excluded because it was not stable enough. The remaining three reference genes were used for normalization (M < 0.5 and pair-wise coefficient variance < 0.15) of the expression data using the qbase+ software (Biogazelle).

### ELISA

The leaf samples originated from the same plants analysed for transgene expression. Approximately 10 mg of freeze-dried leaf material was ground using a FastPrep-24 Instrument (MP Biomedicals, Inc.) and homogenized in 1.5 ml of PBST-buffer (pH 7.4). After centrifugation the supernatants were diluted 1:50 with PBST-buffer. Standards were prepared using freeze-dried Cry1Ab toxin (M. Pusztai-Carey, Case Western Reserve University) identical with the Bt-protein expressed in the Bt maize plants. Thirteen Cry1Ab concentrations were used for the calibration curve ranging from 0 to 6.8 ng/ml dissolved in PBST-buffer. In addition 3 standards were prepared with control leaf extracts from conventional maize. The level of Bt protein in maize leaves was determined using the commercial double antibody sandwich (DAS) ELISA kit (Agdia). Standards and controls were added to a 96-well ELISA microplate in duplicates, samples were added in triplicates. The colour development was measured in a kinetic mode at 650 nm using a Bio-Tek Synergy HT multi detection microplate reader. The colour reaction was stopped after 16 minutes by adding 3 M sulphuric acid and colour intensity was read at 450 nm.

### Statistical analysis

Due to non-normal distribution, the transgene expression data (5 plants per variety and treatment) and the Bt content data (4–5 plants per variety and treatment) were log10 transformed. Three-way ANOVA was used to test for the effects of the variety, stress treatment and the timing of the sampling (before and during stress) on the transgene expression and Bt content. Fold change in the transgene expression and the Bt content between the first (before stress) and the second (during stress) sampling was calculated as the ratio of the ‘during stress’ value to the ‘before stress’ value. Due to non-normal distribution the fold change data were log10 transformed. The fold change means obtained for different stress treatments and plant varieties were compared using Tukey’s HSD method. Correlation between the transgene expression and the Bt protein content was analysed using Spearman rank correlation coefficient (Rs), as recommended by Ponnala et al. [16]. All statistical analyses were performed in JMP 10.0.0 (SAS Institute Inc. 2012).

## Results

### Transgene expression

**Fig 1:**
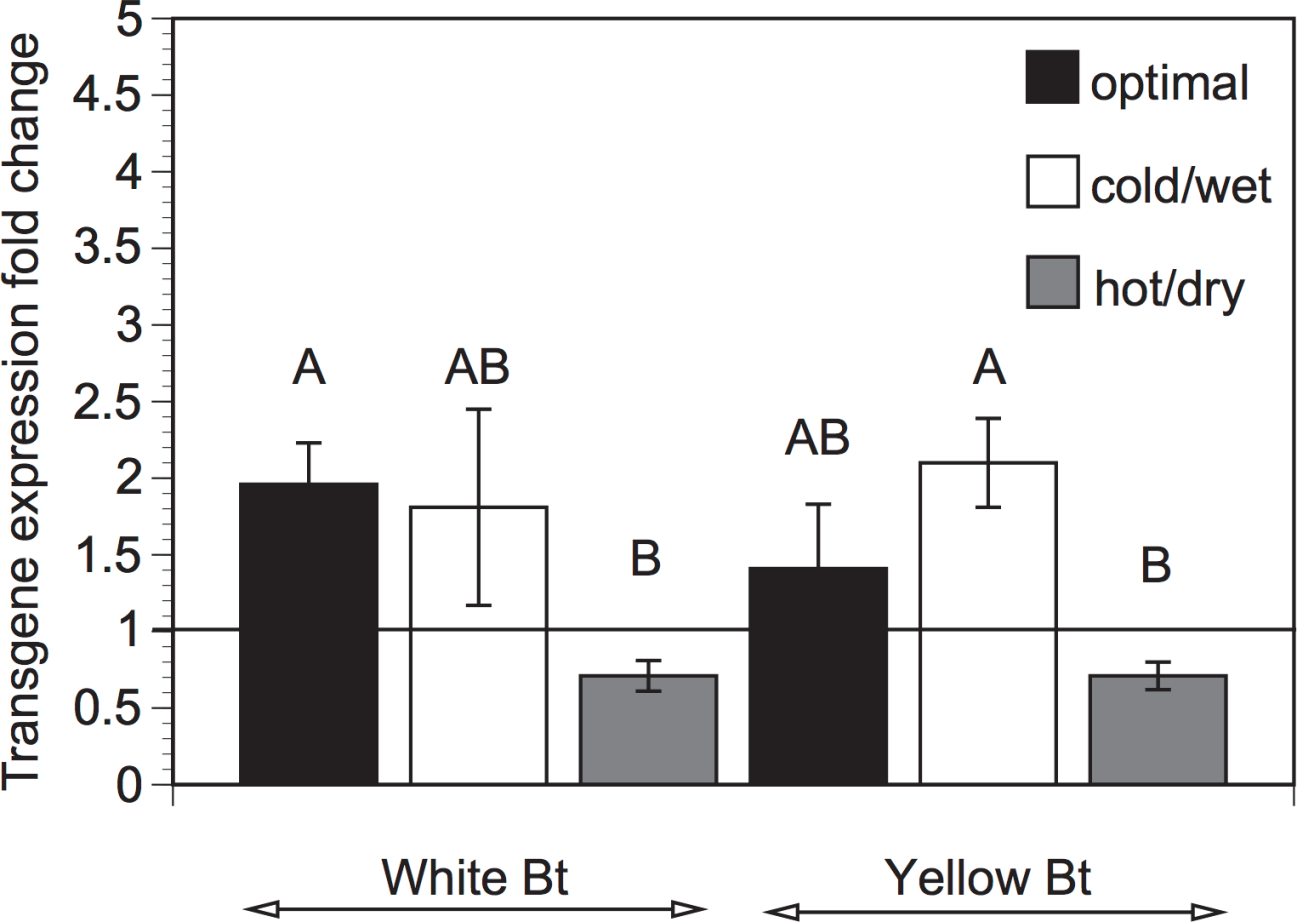
Transgene expression. Fold change in the transgene expression in the upper leaves of the white and yellow Bt maize between the first (before stress) and the second (during stress) sampling. Calculated as the ratio of the ‘during stress’ value to the ‘before stress’ value (i.e. 1 = no change). Plants grown under optimal growth conditions were exposed to no stress. The means labelled with the different letters are significantly different at *P < 0*.*05*, Tukey’s HSD. Means ± SE, n = 5.

There was no significant difference in *cry1Ab* expression in the upper leaves of the two Bt maize varieties (Table 1). Also, the transgene expression did not differ between the treatments (Table 1). We also compared how transgene expression changed during the stress relative to the level before stress (i.e. fold change) in the same plants (Fig 1). In the white Bt maize, the transgene expression under cold/wet stress was similar to the expression under optimal conditions, but was significantly reduced under hot/dry stress. In the yellow Bt maize, the transgene expression under cold/wet and hot/dry stress was not significantly different from the expression under optimal conditions (Fig 1).

**Table 1:** Effects of variety (white Bt, yellow Bt), stress treatment (optimal, cold/wet, hot/dry), sampling time (before and during stress) and their interactions on *cry1Ab* transgene expression and Cry1Ab protein content.

ANOVA		*cry1Ab* transgene expression		Cry1Ab protein content	
	*df*	*F ratio*	*P*	*F ratio*	*P*
Variety	1	0.02	0.889	**5.33**	**0.025**
Treatment	2	0.58	0.561	**5.70**	**0.006**
Sampling time	1	3.17	0.081	1.36	0.249
Treatment*Variety	2	0.11	0.893	0.14	0.866
Treatment*Sampling time	2	**7.41**	**0.002**	1.70	0.195
Sampling time*Variety	1	0.02	0.899	3.66	0.062
Treatment*Sampling time*Variety	2	1.10	0.341	3.10	0.054

Significant effects (*P < 0*.*05*) are shown in bold.

### Bt protein content

**Fig 2:**
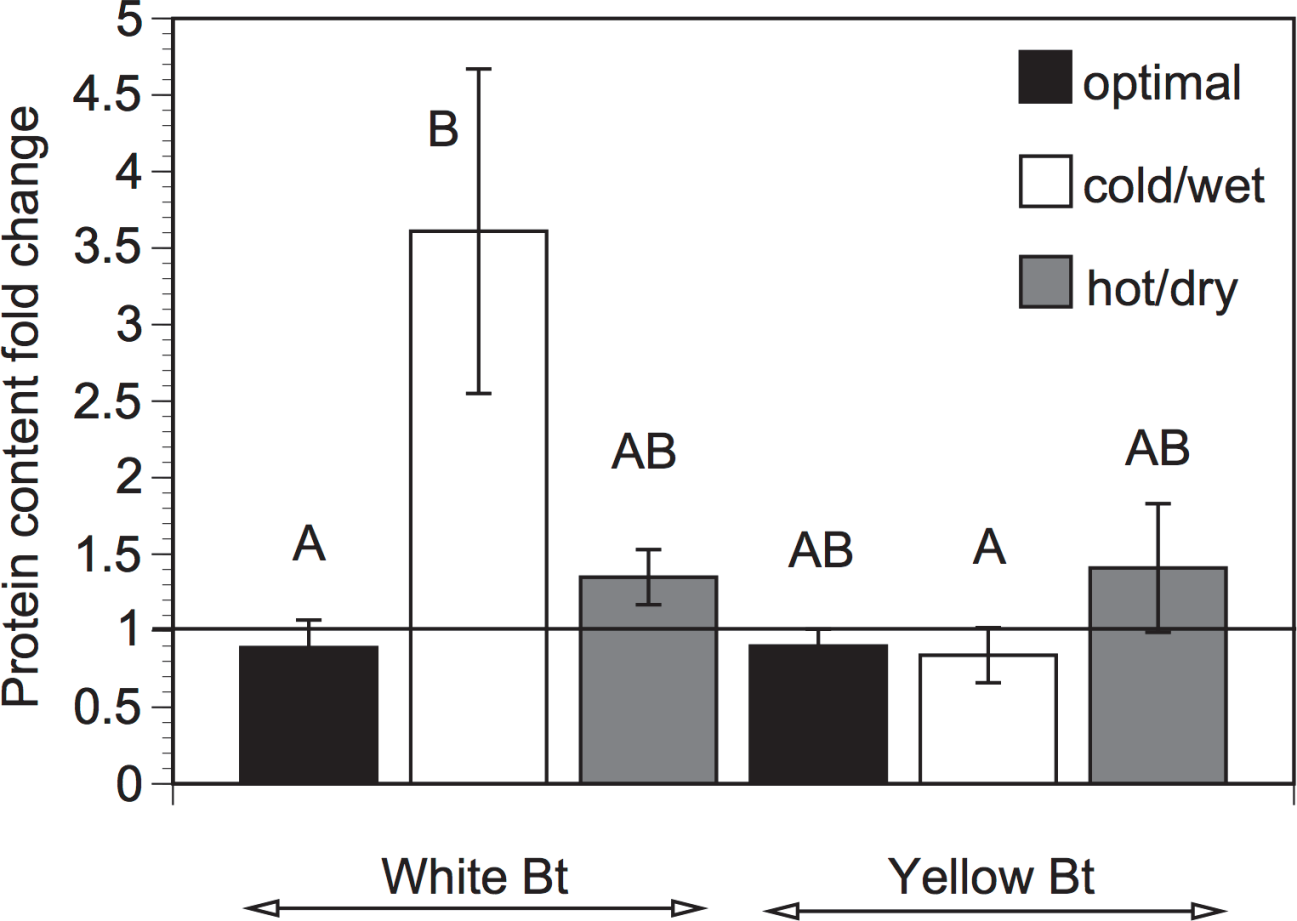
Bt protein content. Fold change in the Bt protein content in the upper leaves of the white and yellow Bt maize between the first (before stress) and the second (during stress) sampling. Calculated as the ratio of the ‘during stress’ value to the ‘before stress’ value (i.e. 1 = no change). Plants grown under optimal growth conditions were exposed to no stress. The means labelled with the different letters are significantly different at *P < 0*.*05*, Tukey’s HSD. Means ± SE, n = 4–5.

There were significant differences in the Bt content in the upper leaves of the two Bt maize varieties (Table 1). The Bt content in the leaves of the yellow Bt maize plants was on average higher than in the leaves of the white Bt maize plants. There were also significant differences in the Bt content between the treatments (Table 1). The Bt content was similar in the plants grown under optimal and hot/dry conditions. However, the leaves of Bt maize plants exposed to cold/wet stress had significantly higher Bt content than the leaves of the plants grown under optimal conditions. When comparing how Bt content changed during the stress relative to the level before stress (i.e. fold change), the Bt content in the white Bt maize plants exposed to the cold/wet conditions increased 4-times compared to the plants grown under optimal conditions, but this was not the case for the same treatment with the yellow Bt maize (Fig 2).

### Correlation between transgene expression and Bt protein content

**Fig 3:**
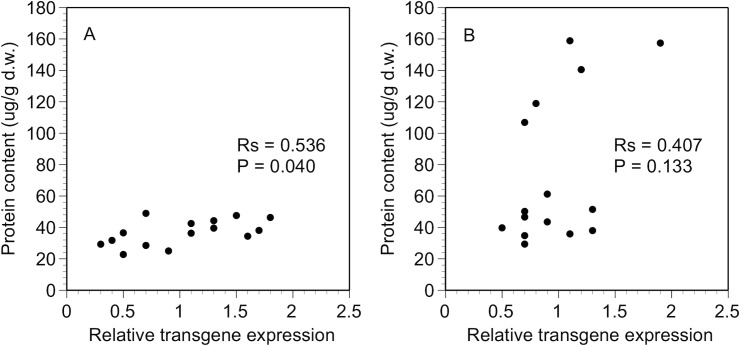
Correlation under optimal conditions. Spearman rank correlation between relative transgene expression and Bt protein content in the plants grown under optimal conditions: A) white Bt maize and B) yellow Bt maize.

The relationship between transgene expression and Bt protein content differed between the two Bt maize varieties. The Bt protein content was correlated with *cry1Ab* transgene expression in the white Bt maize plants (*Rs* = 0.536, *P* = 0.040) but not in the yellow Bt maize plants (*Rs* = 0.407, *P* = 0.133) (Fig 3). Furthermore, the correlation was only found in the white Bt maize grown under optimal conditions before any stress treatment was applied. No correlation between *cry1Ab* transgene expression and Cry1Ab protein content was found in the plants exposed to cold/wet or hot/dry stress (S1 Fig).

## Discussion

In this study we investigated whether there is a correlation between transgene expression and Bt protein content in two Bt maize varieties containing the same transgene cassette (MON 810) and how this relationship is influenced by stressful environmental conditions. Overall, we found no differences in transgene expression between the two different Bt maize varieties which is in accordance with other studies measuring *cry1Ab* transgene expression. La Paz et al. [17] found no significant differences in *cry1Ab* mRNA levels in the leaves of 28 commercial Bt maize varieties. Also, Coll et al. [18–20] reported that mRNA levels of *cry1Ab* were similar between different Bt maize varieties. Thus, our results add to the emerging picture that expression of the transgene *cry1Ab* is not influenced by the genetic background in a significant way.

Simultaneously to quantifying transgene expression, we also measured Bt protein levels in the same plant tissues of the two Bt maize varieties. Despite their similar transgene expression, Bt protein levels differed significantly in the tissue samples of the two Bt maize varieties. The yellow Bt maize leaves contained on average 40% more Bt protein than the white Bt maize leaves. This suggests that mRNA levels of the *cry1Ab* transgene do not necessarily predict its protein, i.e. Bt toxin, level. The relationship between mRNA and protein abundances has been reported to be weak also for native genes [21,22]. Various regulatory processes, including post-transcriptional, translational and protein degradation regulation, occurring after mRNA is made, control native protein abundances [23]. Inhibited protein synthesis, degradation and/or remobilization (or transportation) to developing plant parts have been suggested to cause observed reductions in the amount of Bt protein in transgenic Bt cotton [24]. Similar processes are presumably involved in the regulation of Bt protein content in Bt maize. However, to measure mRNA and protein degradation rates is technically challenging and has not yet been fully explored in plants [16].

Adamczyk et al. [10] reported that *cry1Ac* mRNA transcript levels correlated with Cry1Ac protein levels in Bt cotton. We also found a correlation between *cry1Ab* transgene expression and Cry1Ab protein content, but only in white Bt maize plants. The fact that the correlation between transgene expression and protein content was found in one Bt maize variety and not in the other suggests that other factors also influence Bt protein content. It also shows that Bt protein content cannot be reliably predicted by measuring only mRNA transcript levels. Our results therefore do not support the conclusion of Adamczyk et al. [10].

To investigate how stressful environmental conditions influence the relationship between transgene expression and Bt content, we exposed plants of both Bt maize varieties to cold/wet and hot/dry treatments. Transgene expression under cold/wet stress was similar to the expression under optimal conditions, but the expression of the transgene was reduced under hot/dry stress, though this was only significant in white Bt maize. Also Meyer et al. [25] observed reduction in transgene expression (i.e. a reduction in flower coloration) in transgenic petunia after the plants were exposed to high temperatures. The white flowering plants showed hypermethylation of the 35S promoter directing the transgene expression, in contrast to the fully red flowering plants showing no methylation of the 35S promoter. As *cry1Ab* transgene expression in Bt maize is also driven by the CaMV 35S promoter, it is possible that methylation of the promoter might play a role in the reduced transgene expression under hot/dry conditions.

The reduction in transgene expression under hot/dry conditions did not result in a corresponding, systematic effect on the Bt protein concentration. Also, in the white Bt maize plants the transgene expression under cold/wet stress was similar to optimal conditions, but the Bt content under cold/wet stress increased 4-times compared to optimal treatment. Thus, while transgene expression was correlated to Bt protein content in the white Bt maize under optimal conditions, this correlation was disrupted under stressful conditions. Indeed, during acute stress and developmental changes involving significant proteome remodelling, mRNA-protein correlations are often weaker, with either mRNA or protein lagging in abundance response [16]. Similarly, Li et al. [11] showed that *cry1Ac* mRNA transcript levels in Bt cotton increased in plants exposed to NaCl stress but NaCl treatment did not affect the corresponding Bt protein content in the leaves or roots. In essence, this suggests that under stressful environmental conditions, transgene expression is only a proxy for determining whether the transgene product—here Bt protein—is present or absent and that the Bt protein content is affected by the plant’s own regulatory system and by outside environmental conditions.

Our findings challenge the general presumption that transgenes in commercially approved genetically modified plants are almost invariably expressed at high levels in all plant tissues and phenological phases [3]. We found large variation in the transgene expression and Bt protein content caused by plant genetic background and environmental conditions. Field-grown Bt maize plants might therefore not always produce high enough dose of Bt protein to kill the intermediate (heterozygous) resistant insect pests. Survival of such intermediate resistant pest species on individual Bt plants could increase the probability of resistance development to Bt protein [26]. Moreover, changes in Bt plant efficacy might be mediated through modification of the plant physiological background without any changes in Bt transgene expression and/or Bt protein content [27]. Thus, any assessment of transgenic Bt plants will be incomplete without measuring transgene expression in conjunction with Bt protein content and efficacy.

## Supporting Information

S1 Fig Correlation under stressful conditions. Correlation between relative transgene expression and Bt protein content during cold/wet or hot/dry stress: A) in the white Bt maize and B) in the yellow Bt maize plants. Plants grown under optimal growth conditions were exposed to no stress.
(TIF)

S1 Table Sequences and amplification efficiencies of the TaqMan primers and probes for the reference genes. (PDF)
