# Supplementary figures and images for: Transgene Expression and Bt Protein Content in Transgenic Bt Maize (MON810) under Optimal and Stressful Environmental Conditions
Source: PLoS One. 2015 Apr 8;10(4):e0123011. doi: 10.1371/journal.pone.0123011 (PMC4390241; doi:10.1371/journal.pone.0123011)

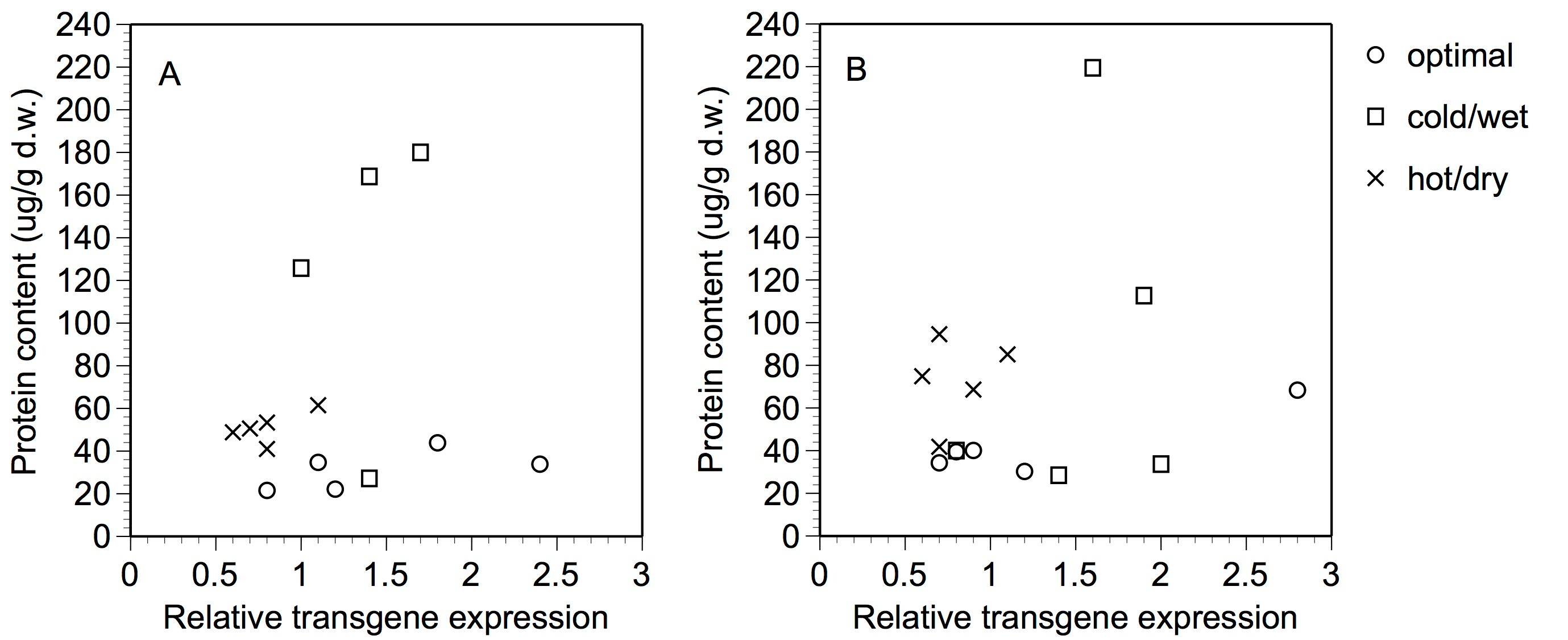

Supplement: S1 Fig — Correlation between relative transgene expression and Bt protein content during cold/wet or hot/dry stress: A) in the white Bt maize and B) in the yellow Bt maize plants. Plants grown under optimal growth conditions were exposed to no stress. (TIF) [file pone.0123011.s001.tif]
