# Supplementary material for: Transgene Expression and Bt Protein Content in Transgenic Bt Maize (MON810) under Optimal and Stressful Environmental Conditions
Source: PLoS One. 2015 Apr 8;10(4):e0123011. doi: 10.1371/journal.pone.0123011 (PMC4390241; doi:10.1371/journal.pone.0123011)
Supplement: S1 Table — (PDF) [file pone.0123011.s002.pdf]

Name	Sequence	PCR efficiency
lug		1.8
for	5’-GAAAACACACGAGTGGAATTGATT-3’	
rev	5’-CGGTCAGAATATGGTCATTCAGTT-3’	
probe	5’ FAM-CGCTGCGATTCAATTCATGCACAAA-TAMRA3’	
mep		2.0
for	5’-CCCACTGGGTTGCAATCCT-3’	
rev	5’-GGTGAGAGCGGAAAGCTTGT-3’	
probe	5’ FAM-ACCCAGACAGATGGCCACCCAACAT-TAMRA3’	
ubcp		1.7
for	5’-ATCCTCTTGTCCCTGAGATTGC-3’	
rev	5’-AGCGTGCGGTGGACTCATA-3’	
probe	5’ FAM-CACATGTACAAGACCGACAGGGCCAA-TAMRA3’	
